# Implantable Pressure-Sensing Devices for Monitoring Abdominal Aortic Aneurysms in Post-Endovascular Aneurysm Repair

**DOI:** 10.3390/s24113526

**Published:** 2024-05-30

**Authors:** Nuno P. Silva, Bilal Amin, Eoghan Dunne, Niamh Hynes, Martin O’Halloran, Adnan Elahi

**Affiliations:** 1 Translational Medical Device Lab, University of Galway, H91 TK33 Galway, Ireland; bilal.amin@universityofgalway.ie (B.A.); eoghandonncha.dunne@universityofgalway.ie (E.D.); martin.ohalloran@universityofgalway.ie (M.O.); 2 Electrical and Electronic Engineering, University of Galway, H91 TK33 Galway, Ireland; 3 School of Medicine, University of Galway, H91 TK33 Galway, Ireland; 4 Western Vascular Institute, Galway Clinic, Doughiska Road, H91 HHT0 Galway, Ireland; niamh.hynes@universityofgalway.ie

**Keywords:** abdominal aortic aneurysm, endovascular aneurysm repair, post-EVAR surveillance, implantable pressure sensor

## Abstract

Over the past two decades, there has been extensive research into surveillance methods for the post-endovascular repair of abdominal aortic aneurysms, highlighting the importance of these technologies in supplementing or even replacing conventional image-screening modalities. This review aims to provide an overview of the current status of alternative surveillance solutions for endovascular aneurysm repair, while also identifying potential aneurysm features that could be used to develop novel monitoring technologies. It offers a comprehensive review of these recent clinical advances, comparing new and standard clinical practices. After introducing the clinical understanding of abdominal aortic aneurysms and exploring current treatment procedures, the paper discusses the current surveillance methods for endovascular repair, contrasting them with recent pressure-sensing technologies. The literature on three commercial pressure-sensing devices for post-endovascular repair surveillance is analyzed. Various pre-clinical and clinical studies assessing the safety and efficacy of these devices are reviewed, providing a comparative summary of their outcomes. The review of the results from pre-clinical and clinical studies suggests a consistent trend of decreased blood pressure in the excluded aneurysm sac post-repair. However, despite successful pressure readings from the aneurysm sac, no strong link has been established to translate these measurements into the presence or absence of endoleaks. Furthermore, the results do not allow for a conclusive determination of ongoing aneurysm sac growth. Consequently, a strong clinical need persists for monitoring endoleaks and aneurysm growth following endovascular repair.

## 1. Introduction

Abdominal aortic aneurysms (AAAs) can be defined clinically as dilation of the aorta artery to 1.5 times greater than its normal diameter (3 cm), affecting predominantly the section of the aorta running through the abdominal region [1,2]. Over time, the walls of the aorta begin to weaken and bulge outwards due to pressure caused by blood. Untreated AAAs frequently rupture, causing catastrophic internal bleeding with a mortality rate between 60 and 80% [3,4,5]. AAA treatment involves the use of grafts to divert the flow of blood into the graft, and away from the disease portion of the aortic wall; this reduces pressure in the aneurysm sac and, consequently, in the weakened wall of the artery. Grafts are used in both open surgery and endovascular aneurysm repair (EVAR) treatments [2].

EVAR is a promising minimally invasive technique which deploys a stent graft in the aneurysmal region by catheter, and is less invasive than open surgery. In addition to the positive outcomes of EVAR, some complications have been reported previously with this technique. These complications include (i) graft migration or graft failure and (ii) endoleaks, which defines the return of the blood flow through the aneurysmal sac [1,2].

Endoleaks appear in 12–44% of cases. Surveillance techniques are required post-EVAR treatment to follow up with patients during their lifetime. Duplex ultrasound (DUX) is the standard surveillance imaging modality with computed tomography angiography (CTA) or magnetic resonance angiography (MRA) performed at 5 year intervals or earlier if the aortic sac is determined to be growing upon duplex surveillance [6,7,8]. However, these cross-sectional imaging modalities have limitations such as high cost in terms of time and resources (burdening the health system), and in the case of CTA, require the patients to be exposed to ionizing radiation and nephrotoxic contrast agents to obtain a quality image of the aneurysm [8,9].

Wireless implantable medical sensors have emerged over the last decades with the aim of substituting current clinical practices to improve the quality of life of patients and possibly allow the remote monitoring of physiological parameters from their homes [10,11,12,13,14]. The implantable sensing technologies have also been trialed for the surveillance of patients post-EVAR. These surveillance technologies include intra-sac pressure-sensing devices to relate the aneurysm sac blood pressure with the presence of endoleaks [7,15]. However, some studies indicate the low effectiveness of the implantable pressure sensors and suggest that the aneurysm size and its growth rate are the best predictors of aneurysm rupture and, consequently, the best indicators for monitoring in post-EVAR surveillance [7,16,17].

Despite the emergence of pressure-sensing technologies for post-EVAR follow-up, discussion remains to adopt new technologies that can be an alternative to the current EVAR surveillance [18]. While numerous studies have reviewed traditional imaging-based surveillance methods, no previous study has consolidated and analyzed findings from both pre-clinical and clinical studies on pressure-sensing devices for AAA post-EVAR [19,20,21]. Therefore, the focus of this study is the review of the current state of the art in post-EVAR surveillance, with a specific focus on pressure-sensing technologies as a potential solution to monitor the aneurysm after the EVAR procedure. This review provides context by summarizing the clinical background of AAA, including current practices in diagnosis, treatment, and surveillance. The main objective of the review is to analyze the literature on commercial pressure-sensing devices designed for post-EVAR surveillance, examining various pre-clinical and clinical studies that assess their safety and efficacy in detecting endoleaks after EVAR procedures. Furthermore, this review also explores common clinical predictors of aneurysm rupture used in traditional imaging-based surveillance methods, which could inform the development of new post-EVAR surveillance devices. Through this comprehensive review and analysis, this review provides valuable insight into the evolving landscape of AAA surveillance and highlights the opportunity to develop new sensing technologies that can address the limitations of the adoption of current pressure-sensing devices.

The remainder of this paper is structured as follow: Section 2 introduces the clinical background of abdominal aortic aneurysms, including epidemiology and pathophysiology of the disease. Then, Section 3 describes the current diagnosis and treatment of AAA. Section 4 defines the current clinical practices in EVAR surveillance and an overview of the current status of alternative surveillance solutions including pressure-sensing devices. Finally, Section 5 and Section 6 finalize the paper, discussing and concluding the most relevant findings.

## 2. Clinical Background

The risk of AAA rupture increases as the diameter of the aneurysm enlarges. Knowledge of the incidence, the risk factors, and the physiological process causing AAA are relevant to prevent aneurysm rupture and, consequently, patient fatality.

### 2.1. Epidemiology

AAAs are considered in the 15 top diseases in the United States, the United Kingdom, and many European countries. The condition has high mortality rates. Approximately, 8000 deaths in the UK and 15,000 in the USA are reported every year [2,22]. The condition is particularly prevalent in people aged over 65 years old and prevalent in 4–8% of that population, predominantly in males [2,23]. Environmental and genetic risk factors are also associated with AAA development as it will be further discussed.

#### Risk Factors

Risk factors are inherent in the development, expansion, and rupture of aneurysms. AAAs are a disease with a high incidence in elderly males. The risk of development is increased by 40% every 5 years after the age of 65; four times higher in males than in females [3,24,25,26]. Endocrine studies reveal that the prevalence of AAAs considering the gender differences might be influenced by hormonal factors that affect the connective tissue of the aorta artery [27,28,29,30]. Despite the low prevalence of AAAs in females, their prognosis is worse than in males, revealing a high mortality rate even after treatment [31,32,33]. Tobacco has a strong clinical association with the development and expansion of AAAs [3,34,35,36]. Studies indicate that the aneurysm growth rate is higher in smoking individuals than in non-smokers [37,38]. Other risk factors associated with AAA are hypertension and obesity [4,39]. However, no strong correlation of these other risk factors with the development of AAA has been found [38]. Additionally, AAAs are more common in patients with atherosclerosis and with a positive family history [35,37,38].

The risk factors associated with the rupture of the aortic aneurysm are overlapped with those associated with AAA development [39]. Aneurysm size is one of the strongest predictors of rupture, with risk increasing greatly at aneurysm diameters greater than 5.5 cm [40,41]. The five-year overall cumulative rupture rate of incidentally diagnosed aneurysms is 25–40% for aneurysms larger than 5.0 cm, compared with 1–7% for aneurysms 4.0 to 5.0 cm in diameter [4,37,39,42]. Smaller AAAs (<55 mm) have exhibited a low frequency of rupture and aortic-related deaths under 1%, yet they have a high overall mortality rate (9.6%). In contrast, larger AAAs (>55 mm) have demonstrated a markedly increased likelihood of rupture and aortic-related mortality of around 25%, alongside a significant overall mortality rate (61.8%). Leone et al. emphasize the necessity for stronger evidence from the risk factors associated with AAA rupture, especially focusing on age, gender, and surgical suitability [43]. The expansion rate may also be a predictor of rupture; a small aortic aneurysm that expands by 0.5 cm or more over a six-month period is considered to have a high risk for rupture.

After aneurysm repair, if the blood re-flows through the aneurysm sac (i.e., endoleak), the risk of aneurysm rupture increases [44]. The blood increases the pressure on the fragile aortic aneurysm wall until the aortic aneurysm ruptures. Also, graft migration or graft failure are risks of aneurysm rupture post-treatment [45,46].

### 2.2. Pathophysiology

The aorta artery is composed of three different layers that are responsible for contributing to the strength and elasticity of the aorta wall. The inner layer (tunica intima) allows the blood flow through the artery. The middle layer (tunica media) is constituted of elastin, muscle tissue, and collagen. Elastic fibers and collagen are responsible for the maintenance of the mechanical and viscoelastic properties of the aorta. The outer layer (tunica adventitia) anchors the aorta in place [2,47,48].

The pathophysiology of the development of AAAs is characterized by a degradation of the elastic fibers, associated with the risk factors described. This elastic degradation reflects the loss of the viscoelastic structure of the artery, increasing the wall stress until the artery ruptures [2,47,48,49]. Additionally, inflammation and vascular smooth muscle cell (VSMC) apoptosis is an indicator of the development and rupture of AAAs [50]. The inflammation process due to the degradation of viscoelastic biocomponents activates the macrophagic response, suggesting a progression of AAA [1,2,23]. VSMC apoptosis is another pathological event related to the AAA progression. However, it is not entirely clear why muscle cell apoptosis occurs as a response to tissue deterioration in AAAs [2,23,51].

## 3. Diagnosis and Treatment

The diagnosis of AAA is usually found during a routine medical test; AAAs are initially an asymptomatic disease [52]. The most common test used to diagnose AAA is an abdominal ultrasound. However, the condition can be diagnosed by CTA or MRA. These two image scans are used before any treatment to detect the size and shape of the aneurysm [1,2].

The AAA treatment type (open surgery or EVAR) is considered for each patient. Guidelines have been designed to support physicians in carefully formulating a plan of the best treatment option for the patient [6]. The aneurysm treatment is associated with low rates of morbidity and mortality.

**Figure 1:**
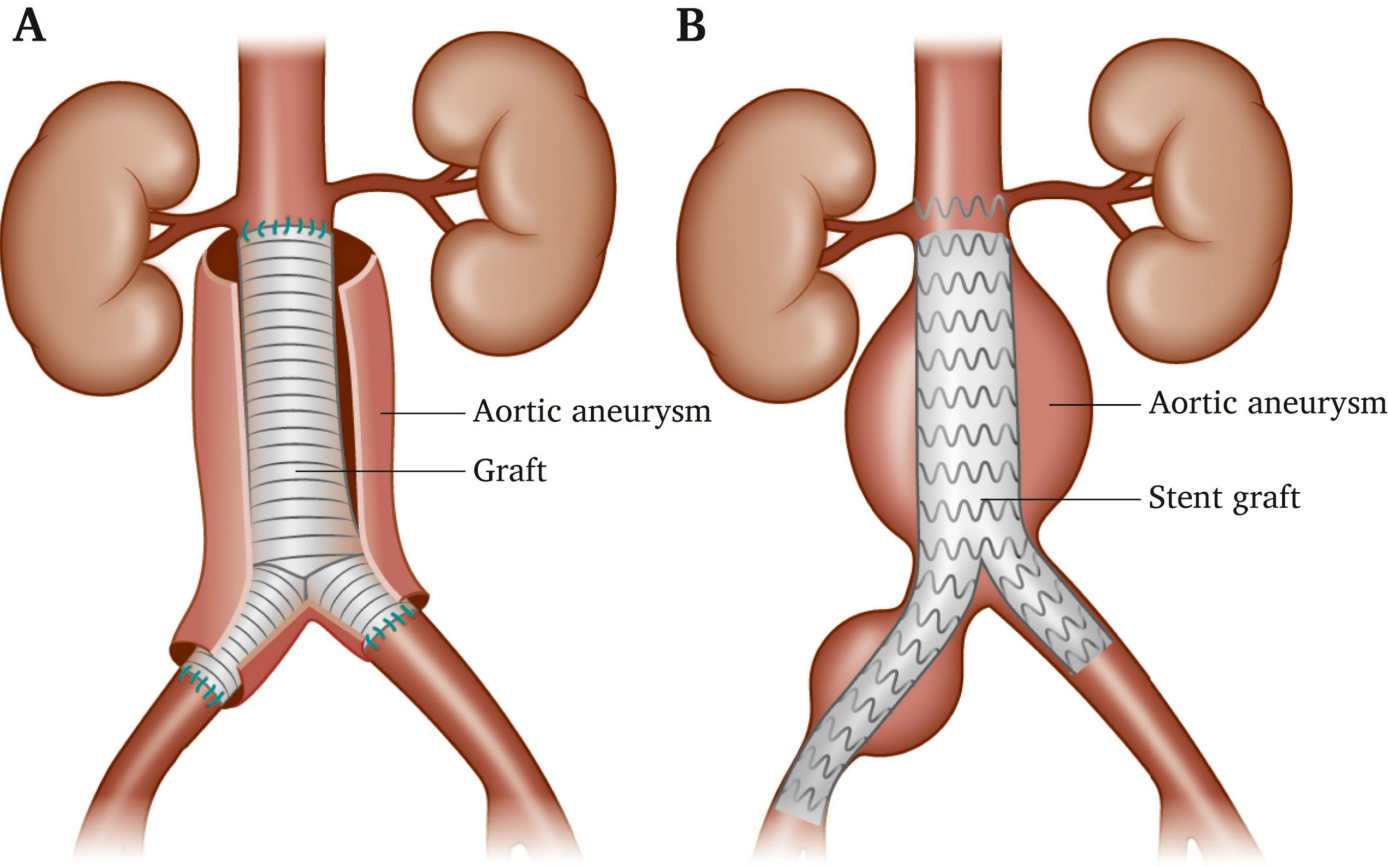
(**A**) Open surgery for an abdominal aortic aneurysm (open AAA repair). The affected segment of the aorta is replaced with a material graft stitched in place. (**B**) Endovascular AAA repair (EVAR). A stent graft is placed inside the aneurysm to reline the aorta and prevent the aneurysm from bursting. Reprinted from publication [6]: copyright (2021), with permission from Elsevier.

Open surgery repair involves removing the aneurysm section of the aorta and replacing that section with a tube graft as shown in Figure 1. The procedure involves opening the aneurysm and sewing a graft into the proximal and distal portions of the aorta. The aneurysmal sac is then closed around the graft. The purpose of the graft is to relieve pressure from the weakened aortic wall, which is closed around the graft [6]. Patients who are treated by open surgery are expected to have a ward recovery of 5–7 days.

Despite the advances in aneurysm repair techniques, mortality following repair remains high, especially in the repair of ruptured aneurysms [2,53]. In contrast to open surgery repair, EVAR is a minimally invasive procedure used to attach a stent graft in the aneurysm region. The stent graft itself is a woven polyester tube supported by a mesh of tubular metal that can expand into the diseased aorta. The edges of the stent graft have barbs and hooks to secure its fastening to the walls [54]. The graft is placed by a catheter inserted through the femoral artery to reach the aneurysm. The graft is then expanded inside the aorta and fastened in place to form a stable channel for blood flow as seen in Figure 1. This technique aims to exclude blood flow from the aneurysm sac in order to remove the pressure from the aneurysm wall [1,2]. Complications due to the physiological stress of open surgery, as well as the subsequent cardiovascular, pulmonary, and renal morbidity are higher in open surgery than in EVAR.

Advancements in the endovascular field have impacted the approach to managing AAA, over the last two decades. The effectiveness of endovascular repair versus open surgical repair has been evaluated in numerous studies [6,55,56]. EVAR presents an initial survival benefit, attributed to a reduced risk of infections and complications related to surgery as shown in Table 1 [55]. Nonetheless, long-term observations reveal a higher occurrence of graft-related issues and the need for further interventions. EVAR has shown a 79% decrease in mortality and 51% fewer complications compared to open surgical repair [56].

In light of the benefits of EVAR compared to open surgical repair as depicted in Table 1, EVAR necessitates ongoing patient monitoring and a high likelihood of requiring subsequent procedures. The primary complication linked to this method involves the occurrence of endoleaks, graft migration, or graft fractures [8,57].

Graft migration or graft failure can occur due to manufacturer error during graft development, or more frequently due to the procedure, where the fixation zone of the graft is not correct or the sealing of the graft does not occur as expected [58].

**Figure 2:**
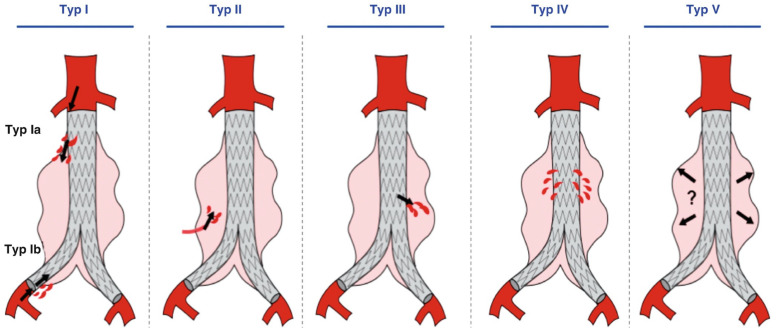
Schematic drawing of the five types of endoleaks after aortic stent graft placement. Reprinted from [61] licensed under Creative Commons CC BY.

Endoleaks are defined as the persistent perfusion of the aneurysmal sac after EVAR. Endoleaks are the main cause of AAA rupture after EVAR [46]. Different types of endoleaks are characterized by their inflow source, regardless of the number and type of other vessels involved in the outflow as observed in Figure 2 [57,59,60]. In Figure 2, the five types of endoleaks are represented, with different interventions indicated for each type [57,61].

The type I endoleak perfuses the aneurysm directly from the aorta (Figure 2 Typ Ia) or iliac arteries (Figure 2 Typ Ib). The flow is originated directly from the endograft seal at the fixation zones. This common type of endoleak occurs in 3% of patients due to device migration or graft kinking. The pressure in the aneurysm sac equals the systematic pressure, leading to a high risk of rupture. This event is led by blood inflow into the aneurysm sac without outflow. The type II endoleak is characterized by a retrograde filling of the aneurysm mainly from other arteries—there is a branch vessel retrograde blood flow. This type of endoleak is the more common one [44]. The type III endoleak is described as an increase in blood flow in the aneurysmal sac due to a structural endograft failure. The risk of aneurysm rupture is much lower in the type II endoleak than in type I and III endoleaks. The graft allows direct communication through the aneurysmal sac. The type III endoleak is also characterized by systematic pressure in the aneurysmal sac. The type IV endoleak is related to the porosity of the graft. The graft allows some blood to pass through the graft to the aneurysmal sac. The type V endoleak corresponds to a continued aneurysm expansion in the absence of a confirmed endoleak [57,61]. For each type of endoleak, its etiology, definition, occurrence and survival rate are further documented and summarized in Table 2.

Li et al. investigated the effects of endoleaks at the time of procedure completion and during subsequent follow-ups on mid-term clinical results. The research offers a detailed examination of the 5-year survival, re-intervention, and rupture rates among patients with or without endoleaks of any type at completion and follow-up. Table 2 presents the survival rates for each endoleak type provided by the study. The analysis further indicates that survival rates are diminished in patients with type I and III endoleaks at follow-up. Moreover, the occurrence of any endoleak during follow-up correlates with reduced chances of avoiding re-intervention and rupture. This evidence underscores the necessity of the follow-up of patients and rigorous monitoring after EVAR, given enduring endoleaks, particularly types I and III [62].

## 4. EVAR Surveillance

The follow-up of post-EVAR patient is crucial for identifying any potential complication arising from the procedure that could increase the aneurysm sac pressure [7,57,63,64].

The current clinical practices for surveillance rely heavily on imaging modalities. While these imaging modalities are effective, imaging can be costly and time consuming, exposing patients to ionizing radiation or contrast agents that may have adverse effects. Furthermore, these modalities do not provide continuous monitoring, which could potentially provide more insights into aneurysm status and allow for early detection of any complications. Implantable sensors have the potential to provide continuous monitoring and improve patient outcomes through the early detection of endoleaks or other complications. In the last two decades, pressure-sensing implantable devices have emerged to assist in post-EVAR surveillance. These medical devices are introduced during EVAR, with the aim of removing the use of imaging to monitor the state of the aorta and to detect complications after repair by pressure measurements [65,66].

While research into new devices aims to decrease reliance on existing clinical methods, the adoption of these devices remains limited. To date, only three devices have been thoroughly researched as potential replacements for existing clinical surveillance techniques. While the aim of this review is to analyze the literature on pressure-sensing devices to evaluate their efficacy in detecting EVAR complications and also explore other parameters that could be targeted for the development of novel sensing technologies, the current clinical practices are described for understanding their advantages and disadvantages, as well as the need of novel solutions for surveillance post-EVAR. Subsequent sections will discuss established clinical surveillance methods for post-EVAR and the newly developed surveillance tools, specifically pressure-sensing devices.

### 4.1. Current Clinical Practices

Currently, EVAR is appropriate for patients with aneurysms larger than 5.5 cm. Patients must be followed up with at 1, 6, and 12 months post-EVAR, and annually afterwards, to detect post-EVAR complications [6,21,67]. Currently, imaging modalities such as CTA, MRA or DUX are used in clinical practice to screen the patients after EVAR procedure, which are cost representative for the health-care system [18,19,68,69]. These techniques have different advantages and disadvantages (see Table 3) for EVAR surveillance [63,70]. In some patients, ultrasonography might be used as a solo modality for surveillance following EVAR since it decreases radiation exposure to the patients and it has low costs for the health system [71].

With CTA, the prolonged nature of post-EVAR surveillance subjects patients to substantial radiation and contrast agent doses [9,72]. The need for a considerable number of scans can expose patients to anaphylactic reactions and nephrotoxicity due to the iodine contrast used in this imaging modality [8]. Furthermore, the current use of ionizing radiation may damage the DNA of cells, leading to possible cancer development [6,9]. However, no radiation is used in MRA, and the contrast agent is considered safer than the one used in CTA [9]. DUX does not use any radiation [72]. However, contrast enhanced ultrasound is commonly used for DUX surveillance to enhance endoleaks detection and the type and source of endoleaks. The contrast agent does not present nephrotoxicity like the contrast agents used in CTA. However, this cheap and safe technology is not used due to the lack of reproducibility. Also, this method is operator dependent. Some reports exploit some difficulty of visualization of the abdominal viscera using DUX in some patients, as it is complicated to provide a definitive outcome from the image scan [6,7].

### 4.2. Pressure-Sensing Devices

**Figure 3:**
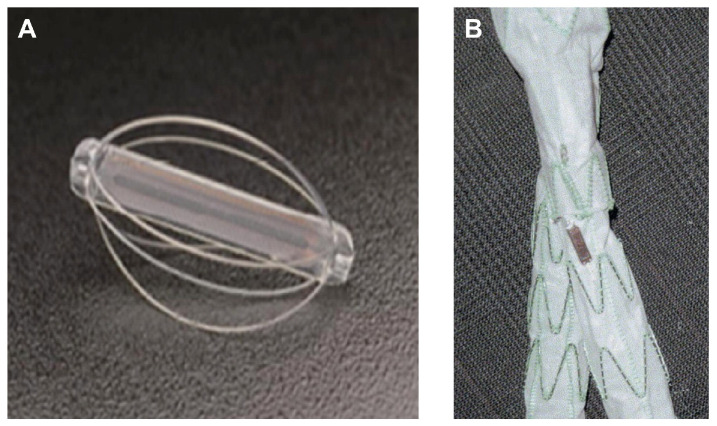
(**A**) EndoSure Wireless AAA Pressure Sensor (CardioMems, Atlanta, GA, USA). Reprinted from publication [60]: copyright (2021), with permission from Elsevier. (**B**) ImPressure AAA Sac Pressure Sensor (Remon Medical Technologies, Caesarea, Israel). Reprinted from publication [73]: copyright (2021), with permission from Elsevier.

Over the last two decades, implantable pressure sensors have been proposed mainly to detect the presence and type of endoleaks after EVAR since certain types of endoleaks are a risk indicator of aneurysm rupture as mentioned previously [7,65,66]. The devices use the principle of pressure sensing in order to provide feedback to physicians regarding the increase in pressure in the aneurysm sac [7,60]. EVAR failure leads to an increase in blood flow within the aneurysm cavity, increasing the pressure within the aneurysm sac. Some studies have linked changes in intra-sac pressures to potential endoleaks [60,73,74,75,76]. To date, three devices using implantable sensors (see Figure 3) to measure pressure in the aneurysm sac have been created [66]. Clinical trials were conducted to understand and classify the effectiveness of these techniques post-EVAR surveillance [77,78,79]. The studies attempted to correlate the intra-sac pressure measurements between the pressure sensor and angiographic measurements [73,80]. Additionally, studies also investigated the correlation of the pressure measured with the sensor and the incidence of endoleaks [59,60,81]. The studies reported for the clinical trials the approval by the Ethics in Research Committee. Additionally, it is reported on the cited work that all patients signed an informed consent form.

Three different sensors were designed to attempt to register the blood pressure values within the aneurysm sac. The measurements then compared the normal blood pressure to correlate with any graft failure or the presence of endoleaks.

#### 4.2.1. ImPressure AAA Sac Pressure Sensor

The ImPressure AAA Sac Pressure Sensor (Remon Medical Technologies, Caesarea, Israel) was proposed to measure the aneurysm sac pressure [73]. This device is sewn on the endovascular graft as shown in Figure 3B.

*Working Principle:* The sensor uses a small transducer that contains a piezoelectric membrane that energizes a capacitor when actuated by ultrasound waves from a handheld probe. Once activated, the piezoelectric membrane generates a signal according to the pressure sensed, which is received by the handheld probe and then converted in pressure values [73,78]. The measurements are conducted by an ultrasound technician that activates the sensor and reads the pressure values at that moment.

Initial trials were carried out on six pigs that had surgically implanted AAA, then were treated with endovascular repair [82]. The objective of this trial was to see whether the sensor implanted within the AAA could accurately and reliably provide pressure measurements, and how durable the sensor was while covered in a large, organized thrombus mass. The pressure was taken by an arterial catheter along with the sensor reading at the time of implantation. After, sensor measurements were taken weekly for two months. Three of the pigs survived for the full eight weeks. All sensors read pressure successfully at each attempt, and all sensors were deeply embedded in the thrombus. The animal trial concluded that the pressure measurements were accurate and reliable according to the baseline pressure values for the animal under test. The experimental trial also concluded that the sensor was sufficiently robust to be maintained during all experiments, even when embedded in a thrombus mass [82].

In the first experimental human trial in 2004, 14 patients underwent the EVAR procedure with an average aneurysm size of 6.3 ± 0.9 cm [73]. The sensor was successfully deployed in the aneurysm sac of all patients. Results showed that in 4 patients, the sensor was unable to obtain pressure measurements from the transducer; in the remaining ten patients, a decrease in pressure after EVAR was observed. From the remaining patients under clinical trial, one was diagnosed with a type I endoleak and three with a type II endoleak. From this clinical trial, it was concluded that successful EVAR procedures were found to have a gradual decrease in pressure throughout the trial. Due to the excellent initial concordance and the regular pressure tests, the sensor was deemed to be accurate. Apart from the good pressure measurements obtained in the trial, any correlation between pressure and presence and type of endoleaks was impossible to make [73].

**Figure 4:**
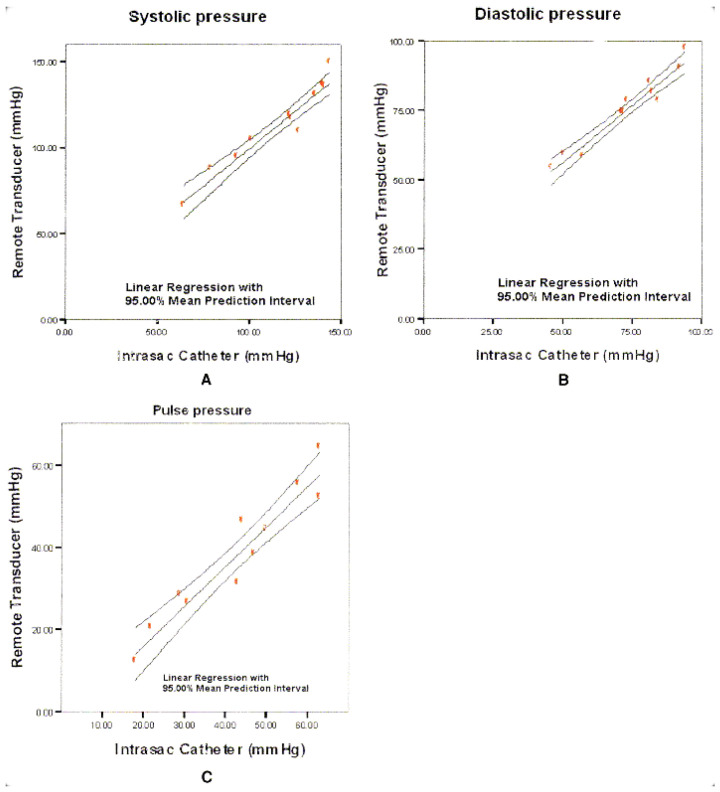
Results obtained from the first clinical study conducted in 14 patients with the ImPressure AAA Sac Pressure Sensor (Remon Medical Technologies, Caesarea, Israel). Graphics represent the pressure measured in the intra-sac catheter and the ultrasound transducer: (**A**) systolic pressure, (**B**) diastolic pressure, and (**C**) pulse pressure. Reprinted from publication [73]: copyright (2021), with permission from Elsevier.

Another clinical trial (2006) was conducted using the same sensor in 21 patients [74]. The sensor was successfully deployed in 20 patients. Pressure measurements were taken from 15 patients for the total number of monthly visits as shown in Figure 4. Five patients were excluded during the study due to the development of endoleaks. This study was not designed to correlate the pressure with the presence of endoleaks. However, it was observed that the pressure was elevated in two patients with type II endoleaks, compared to the ones without the presence of endoleaks. These two patients did not have sac expansion. Shrinkage of the aneurysm was only observed in seven patients, with consequently lower sac pressures [74,83].

In summary, although clinical trials indicated that this sensor may be able to accurately measure sac pressures, the use of an external ultrasound transducer remains a limitation for clinical adoption. Therefore, patients must still go to a medical center to have pressure readings evaluated. Furthermore, the sensor has not received regulatory approval from the FDA.

#### 4.2.2. EndoSure Wireless AAA Pressure Sensor

The EndoSure Wireless AAA Pressure Sensor (CardioMems, Atlanta, GA, USA) (Figure 3A) is a minimal pressure device deployed during the EVAR procedure with approval from FDA [60].

*Working Principle:* This device is composed of two coils of copper wire within a fused silica matrix. The surface of the device is pressure sensitive. The coils change their resonance frequency due to changes in capacitance caused by the surrounding pressure. An external antenna emits multiple radiofrequency pulses that activate these coils and cause them to vibrate. This vibration is received by the external antenna that relates the frequency received to a quantitative pressure [78].

Initial animal trials were carried out in 2006 in four dogs that had surgically implanted AAA. The objective of this trial was to observe the ability to measure pressure in the presence of type II endoleaks. Intraoperative readings were taken with a catheter and the sensor. All measurements showed lower intra-sac pressures when the aneurysm was fully excluded by the graft. When the type II endoleaks were introduced, resulting pressures increased compared to the intra-sac pressures when full exclusion occurred. The sensor was able to accurately measure readings in all animals [81].

In 2007, a clinical trial used this sensor in 12 patients to record their intra-sac pressures at regular intervals. Like many other studies, post-EVAR pressure decreased significantly (average of 33% decrease in all patients). The study concluded that remote sac pressure measurements may be useful in providing care to endoleaks in addition to CTA scans [79].

**Figure 5:**
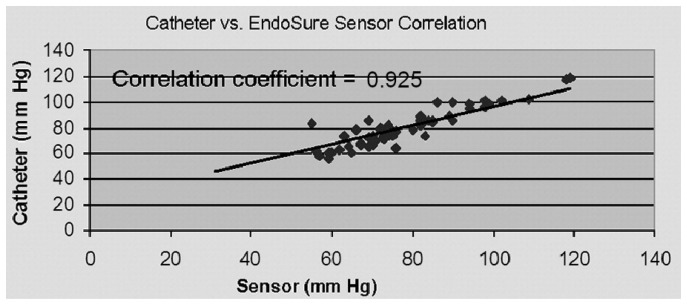
Results obtained from the APEX clinical trial with the EndoSure Wireless AAA Pressure Sensor (CardioMems, Atlanta, GA, USA), and represents the first pressure reading between the sensor and the angiographic catheters in the aneurysmal sac. Reprinted from publication [60]: copyright (2021), with permission from Elsevier.

Initial results from the APEX clinical trial (2007), using this pressure sensor in 90 patients, reported the exclusion of 14 patients due to protocol deviations; hence, no pressure measurements were conducted for these 14 patients [60]. Patients were excluded from the study due to the absence of necessary data, indicating that the sensor failed to capture their pressure readings. The study also notes a learning curve related to the methods of inserting the sensor, reading the sensor, and managing the electronic components. However, in 70 patients, pressure measurements agreed between the sensor and angiographic measurements (as showed in Figure 5), regarding the presence or absence of any endoleak of type I or III. From this sensitivity and specificity study, the overall results showed a sensitivity of 0.94 and a specificity of 0.80 for detecting an endoleak of type I or III, as well as the presence or absence of endoleaks. In this study, the endoleak type I or III detection from the device was correlated with the hypothesis of a less than 30% reduction in pulse pressure. From the patients tested, only a total of five developed an endoleak. A pulse pressure lower than 30% was observed in four of the patients, while the other patient has a pulse pressure higher than 30%. Regarding the sensitivity of the sensor and the attempt to correlate pressure with endoleak type, such was not observed. In fact, the pressure can roughly infer the presence of an endoleak, without identifying its type. Furthermore, on the remaining patients of the study, five patients also showed a pulse pressure decrease of less than 30%; however, no endoleak was present for those patients. Aside from these results, the choice of the percentage threshold indicative of endoleak presence is questionable since no studies were provided to show that limited number. No more literature or clinical trials have been reported since 2007.

#### 4.2.3. Telemetric Pressure Sensor

Another novel technology reported in the literature is the Telemetric Pressure Sensor (Helmholtz Institute for Biomedical Engineering and the Institute of Materials in Electrical Engineering, RWTH Aachen, Aachen, Germany) [84].

*Working Principle:* This pressure sensor uses a digital processing unit in-capsule constituted by a radiopaque coil that transmits data over an inductive link. Moreover, image fluoroscopy scans allow the visualization of the capsule in the body.

To date, no clinical trials have been undertaken [76,84].

### 4.3. Efficacy of the Pressure-Sensing Devices

From preliminary results, all clinical trials with the ImPressure and EndoSure sensors support their efficacy to measure systemic blood pressure and to give the pressure values as an outcome for physicians (Table 4). From Figure 4 and Figure 5, the primary investigations involving the ImPressure and EndoSure pressure sensors demonstrate a strong correlation between the readings obtained from the devices and those measured using an intra-sac catheter. Despite their technological differences, both devices effectively gauge the aneurysm’s pressure.

Also, in animal experiments, both devices showed a reduction in aneurysm intra-sac pressure when the sac was excluded by the graft. However, no direct relation between the pressure measurements and type of endoleak was established [57,78,81]. The clinical trials conducted using these devices had very few patients. Additionally, long-term studies were suggested by different authors to prove the efficacy for post-operative EVAR surveillance [60,78,80]. Moreover, a serious question regarding the cost and effectiveness of pressure-sensing devices is debatable [83].

An investigation of the efficacy of pressure sensors to determine only type II endoleaks observed that none of the clinical studies investigating the efficacy of pressure sensors had enough patients to efficiently determine the long-term usage of the sensor [77]. Further, there is no clarification in the correlation of pressure measurements with the type of endoleak. However, in all clinical trials, a decrease in the blood pressure was observed when the aneurysmal sac was excluded as seen in Table 4. Moreover, the study also highlights the question of whether a single pressure measurement is enough to distinguish and predict the presence of type II endoleak [77,82].

**Figure 6:**
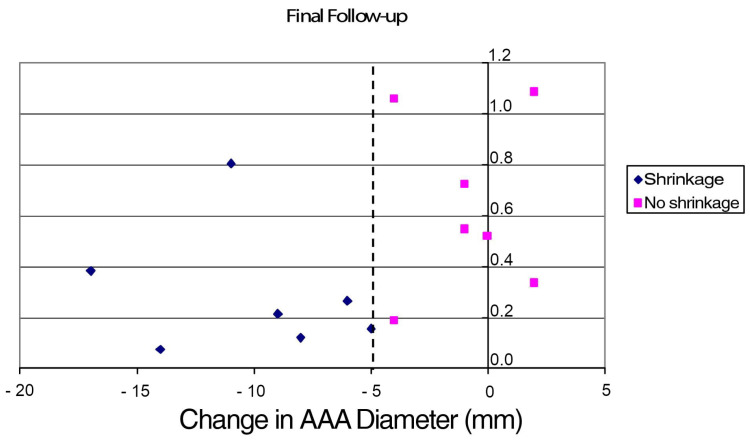
Final follow-up of the post-EVAR surveillance clinical study using the ImPressure AAA Sac Pressure Sensor (Remon Medical Technologies, Caesarea, Israel) in 21 patients. Pressure measurements from the device were plotted with changes of AAA diameter. Diameter was determined by phase contrast-enhanced CTA or MRA, computed from the initial scan and after two years. Reprinted from publication [74]: copyright (2021), with permission from Elsevier.

During the final follow-up of a clinical investigation using the ImPressure AAA Sac Pressure device (Remon Medical, Cesaria, Israel), patients who had undergone the EVAR procedure were assessed to explore the correlation between the pressure measured in the aneurysmal sac and the changes in the AAA diameter. Figure 6 shows the graph derived from this study. The observations indicate that the device did not consistently correlate the intra-sac aneurysmal pressure measurements with variations in the diameter of the aneurysm. Previous studies, such as those cited by Sonesson et al. [75], suggest that lower pressure readings may lead to aneurysm shrinkage. Nonetheless, this particular study did not conclusively establish a direct or inverse relationship between the pressure levels and the size alterations of the AAA. The effort to link pressure readings from implantable devices to changes in aneurysm size highlights the clinical feature that necessitates enhancement for the medical community regarding patient outcomes post-EVAR.

## 5. Discussion

The current clinical practice in post-EVAR uses imaging modalities to visualize possible alterations or endoleaks development. None of these imaging modalities is ideal, as they pose possible risks to the patient such as operator dependence, radiation, or toxicity of the contrast agents. Moreover, those image modalities require trained clinicians or time and cost consumption of health services.

To overcome the limitations of current clinical practices in post-EVAR surveillance, new technologies were developed based on the principle of pressure increase due to blood re-flow of the aneurysm sac (detailed in Table 4). Regarding the working principles of the devices, while the ImPressure sensor uses ultrasound-based technology to actively obtain pressure readings from the sensors, the other two devices investigated use magnetic inductive coupling to communicate from an external device with the implantable sensor. Both technologies under the scope of this sensor are considered safe and low cost. Ultrasound-based technologies have been investigated over the last decades, while magnetic inductive coupling (wireless implantable medical sensors) has been recently investigated to create non-battery implantable devices which can be implantable on the human body [10,12,86]. The aim of the development and optimization of implantable sensors is to create novel cost-effective solutions for the patients and medical community, decreasing the influx of patients to the health care facilities.

The efficacy of these devices was evaluated in clinical trials to correlate aneurysm sac pressure and the presence of endoleaks. In all trials, sensors were able to measure pressure in the aneurysm sac and able to transmit that information to a physician. However, a small number of patients were excluded from the clinical trials because it was unable to obtain pressure measurements readings from the device. Additionally, the implantable pressure sensors were sometimes able to detect the presence of endoleaks but unable to differentiate between endoleak types. An important step forward in the development of post-EVAR surveillance methods requires an efficient and long-term usage device able to detect and characterize endoleaks.

The pressure sensors were able to successfully measure the pressure of the aneurysmal sac; however, there was no evidence that could relate the increase or decrease in the aneurysm size during clinical trials. Additional studies should be conducted in order to observe whether the pressure sensing can determine the growth rate of AAA.

The literature and clinical practice indicate that the aneurysm size and growth rate prior to and post-aneurysm are important indicators for predicting aneurysmal rupture, highlighting the significance of these factors. Considering the fatality of an aneurysm rupture, clinicians perform a follow-up with patients before surgical intervention to understand the growth of the aneurysm [17,85]. Notably, the studies have demonstrated that the risk of aneurysm rupture is closely related to the relative change in the size of the aneurysm. Furthermore, long-term patient surveillance has revealed low rates of morbidity [87], highlighting the importance of surveillance post-EVAR. Therefore, the current medical protocols necessitate follow-up imaging to monitor aneurysm dimensions, facilitating comparison with earlier obtained images from the patient. Such comparisons, which suggest changes in aneurysm size, underscore the importance of measuring these dimensions as a marker of EVAR effectiveness. The detection of aneurysm growth after repair is indicative of the possible presence of endoleaks or other related clinical problems with the graft.

Furthermore, the relationship between aneurysmal rupture risk and aneurysm dimensions has also been investigated through computational modeling. Specifically, studies have employed numerical simulations to quantify the mechanical wall stress induced by variations in aneurysm size and growth rate [88,89]. The cumulative evidence supports the notion that aneurysm size and expansion rate are reliable predictors of aneurysm rupture risk, particularly in the post-EVAR [16,17,58,90]. A recent review study by Vaitenas et al. revealed that measuring the volume of AAA post-EVAR provides good outcomes in predicting endoleaks and the growth of aneurysms, and it also demonstrated more significant correlations with clinical outcomes. However, the studies lacked standardized protocols for measuring both volume and diameter, which limits the interpretation of the results. The study concluded that volume measurement is favored over diameter measurement in assessing abdominal aortic aneurysm (AAA) due to its accuracy in predicting endoleaks and aneurysm growth [91]. However, given the risks associated with current imaging techniques used for AAA monitoring after EVAR, there appears to be a pressing need for a different monitoring approach that employs novel sensing technologies to monitor the alteration in the size and the growth rate of the aneurysm after repair for post-EVAR surveillance.

## 6. Conclusions

One of the major implications of post-EVAR surveillance is the use of CTA or MRA to follow up with patients. The use of radiation and nephrotoxic contrast agents as part of these image modalities is a risk for the patient’s health. Additionally, these image modalities represent high cost and time consumption for health-care systems. The aim of new methods for post-EVAR follow-up surveillance must be to represent an effective, low-cost, and easy-to-use system that is able to clearly identify any complication related to the endovascular repair procedure. Some technologies have been developed to follow up with patients post-EVAR without the use of the current follow-up methods. These emergent technologies are based on implantable pressure sensors, which exploit the correlation between aneurysm sac blood pressure and the presence of endoleaks. Despite some encouraging results, no clear evidence could relate the aneurysm sac blood pressure and the presence of endoleaks.

The three implantable pressure-sensing devices under investigation were able to accurately measure the intra-sac pressure of the aneurysm. However, the devices failed in the correlation of the pressure with the formation of endoleaks or graft-related complications. Some studies have suggested that the best predictors of aneurysm rupture are the aneurysm size and the aneurysm growth rate, or aneurysm volume. Therefore, there is a need for a novel technology that could monitor aneurysm size and aneurysm growth rate without using the imaging modalities and without requiring patients to visit hospitals.

## Figures and Tables

**Table 1:** Advantages and disadvantages of open surgical and endovascular AAA repair.

	Open Surgery Repair	Endovascular Repair
**Invasive**	Yes	Minimally
**Hospitalization**	5–10 days	1–3 days
**Risk**	High surgical complication risks	Fewer major complications
**Follow-up**	-	Regular
**Durability**	Proven Durability	High probability of re-intervention
**Mortality Rate**	<5%	<2%
**1-year Survival**	59%	73%

**Table 2:** Types of endoleaks including etiology, occurrence, survival rate and treatment.

	Type I	Type II	Type III	Type IV	Type V
**Etiology**	Attachment site leaks	Collateral vessel leaks	Graft failure	Graft wall porosity	Endotension
**Definition**	Blood flow into the aneurysm sac at the proximal (Type IA) or distal end (type IB) of the stent attachment sites	Retrograde blood flow into the aneurysmal sac from a branch vessel	Mechanical failure of the stent-graft; leakage of blood through the stent defect	Blood flow through stent-graft porosity	Aneurysm expansion in the absence of a visible leak
**Occurrence [%]**	∼3	20–30	∼2	Rare	Rare
**Survival Rate [%] [62]**	69	79	73	73	75
**Treatment**	Immediate repair: stent graft adjustment	Management with regular surveillance unless there is aneurysm expansion	Immediate repair: covering the defect with a stent extension	Resolved after normalization of the coagulation	Open surgery

**Table 3:** Characteristics of current practice imaging modalities for EVAR surveillance.

	DUX	CTA	MRA
**Detection of EVAR Complications:**			
Aneurysm sac enlargement	Yes	Yes	Yes
Endoleak	Yes	Yes	Yes
Graft migration	Yes	Yes	Yes
Graft infection	Limited	Yes	Yes
**Risks**	None	Ionizing radiation and contrast nephropathy	Risk of nephrogenic systemic fibrosis
**Technical aspects**	Operator and patient dependent	Timing of contrast administration	Unsuitable for ferromagnetic stents and pacemaker bearers
**Suitable as sole modality for EVAR follow-up**	No	Yes	No

**Table 4:** Resume of EVAR surveillance using pressure-sensing devices to monitor AAA post-EVAR procedure.

	ImPressure AAA Sac Pressure Sensor	EndoSure Wireless AAA Pressure Sensor	Telemetric Pressure Sensor
**Parameter**	Pressure	Pressure	Pressure
**FDA approval**	No	Yes	No
**Experimental trials**	6 Pigs; 35 Humans	4 Dogs; 102 Humans	No
**Successful readings**	100%; 71%	100%; 77%	-
**AAA shrinkage**	Yes	Yes	-
**Endoleak detection attempt**	No	Yes	-
**Observations**	Pressure readings took successfully in the animal trial. First human trial (n = 14), it observed a decrease in aneurymal sac pressure. Second human trial (n = 21), pressure measurements were obtained in 15 patients. Observed an aneurysm shrinkage in 7 patients. No relation with presence or absence of endoleaks.	Lower pressure readings when endoleaks type II were introduced in the animals. First human trial (n = 12), it observed an average decrease of 33% in pressure. No relation with presence or absence of endoleaks. Second human trial (n = 90), it tried to relate the pressure measurements with endoleak type I or III. No direct relation was obtained for specific endoleak type.	-
	[73,75,84,85]	[60,80,82]	[76]
